# Application and prospect of the therapeutic strategy of inhibiting cellular senescence combined with pro‐regenerative biomaterials in regenerative medicine

**DOI:** 10.1002/SMMD.20230030

**Published:** 2023-12-21

**Authors:** Qianyi Li, Zhenzhen Wang, Nuo Shi, Yang Qi, Wenfei Yao, Jie Yu, Yiming Lu

**Affiliations:** ^1^ Department of Emergency Ruijin Hospital Shanghai Jiao Tong University School of Medicine Shanghai China; ^2^ Pôle Sino‐Français de Recherches en Sciences du Vivant et G´enomique Shanghai China; ^3^ International Laboratory in Cancer, Aging and Hematology Shanghai Jiao Tong University School of Medicine/Ruijin Hospital/CNRS/Inserm/Côte d'Azur University Shanghai China; ^4^ Peterson's Lab Shanghai China; ^5^ Division of Critical Care Nanxiang Hospital of Jiading District Shanghai China

**Keywords:** aging and regeneration, cell senescence, senolytics and senomorphics, tissue engineering

## Abstract

Complete regeneration of damaged tissues/organs has always been the ultimate challenge in regenerative medicine. Aging has long been considered the basis of age‐related diseases, as senescent cells gradually accumulate in tissues with increasing age, tissues exhibit aging and normal physiological functions are inhibited. In recent years, in damaged tissues, scholars have found that the number of cells with features of cellular senescence continues to increase over time. The accumulation of senescent cells severely hinders the healing of damaged tissues. Furthermore, by clearing senescent cells or inhibiting the aging microenvironment, damaged tissues regained their original regenerative and repair capabilities. On the other hand, various biomaterials have been proved to have good biocompatibility and can effectively support cell regeneration after injury. Combining the two solutions, inhibiting the cellular senescence in damaged tissues and establishing a pro‐regenerative environment through biomaterial technology gradually reveals a new, unexpected treatment strategy applied to the field of regenerative medicine. In this review, we first elucidate the main characteristics of senescent cells from morphological, functional and molecular levels, and discuss in detail the process of accumulation of senescent cells in tissues. Then, we will explore in depth how the accumulation of senescent cells after damage affects tissue repair and regeneration at different stages. Finally, we will turn to how to promote tissue regeneration and repair in the field of regenerative medicine by inhibiting cellular senescence combined with biomaterial technology. Our goal is to understand the relationship between cellular senescence and tissue regeneration through this new perspective, and provide valuable references for the development of new therapeutic strategies in the future.

## Graphical abstract

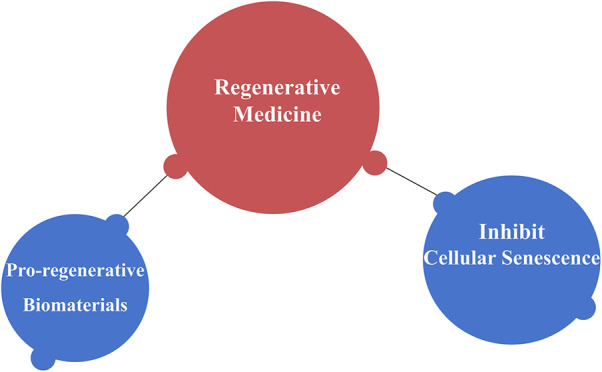

**Key points**

- Emphasized the current status where the accumulation of senescent cells impedes tissue regeneration.
- Clarified that the removal of senescent cells or inhibition of the aging microenvironment enables damaged tissues to regain their original regenerative and repair capabilities.
- Summarized the innovative treatment strategy and its future prospects by inhibiting cellular senescence in damaged tissues and establishing a pro‐regenerative environment through biomaterial technology in the field of regenerative medicine.

## 1 INTRODUCTION

Regenerative medicine is a branch of medicine that involves the use of stem cells, biomaterials, molecular mechanism and engineering technologies to repair, regenerate or replace damaged tissues and organs.^1^ It has undergone significant developments in the past few decades and has gradually become a multidisciplinary field of research. Historically, the origins of regenerative medicine can be traced back to the early research on organ transplantation and tissue engineering in the 1960s and 1970s. However, the real rise of the field was in the 1990s and early 21st century, with the development of stem cell research, in particular the isolation and culture of human embryonic stem cells, and the discovery of induced pluripotent stem cells.^2^ In regenerative medicine, scientists, engineers and physicians utilize knowledge and tools from various disciplines to commit to regenerating and repairing injured or degenerated tissues and organs.^3^ At the same time, this interdisciplinary combination gives regenerative medicine very high potential in medical and biomedical research, and is expected to provide new therapies for many clinically untreatable or difficult‐to‐treat diseases.

Regenerative medicine is an extremely complex field, the success of which depends on an in‐depth understanding and precise control of a series of key factors. Before formulating therapeutic strategies, scientists need to explore and analyze the various factors affecting sustainable tissue regeneration. In recent years, scientists have found that with increasing age, the regenerative capacity of most organisms will gradually decline.^4^ For example, the self‐repair ability of human tissues such as skin, liver and intestines decreases with age. Similarly, some important regenerative cells, such as stem cells, are also affected by aging, with decreased numbers and functions.^5^ Aging and regeneration can indeed be seen in many ways as a contradictory relationship. Some changes during the aging process, such as chronic inflammation, changes in the microenvironment, and accumulation of senescent cells, may affect the regeneration process. These changes may affect cell proliferation, differentiation and migration, thereby affecting tissue regeneration.^6^ However, although aging and regeneration are contradictory in many ways, they are not completely irreconcilable. In scientific research, there have been many studies trying to address the obstruction of aging to regeneration through means such as stem cell technology, gene editing technology, drug intervention, etc., in order to improve the regenerative capacity of the elderly, and even slow down the aging process.^7^ At the same time, these studies have provided valuable information for us to understand the complex relationship between aging and regeneration, and how to use this knowledge to improve human health.

**FIGURE 1:**
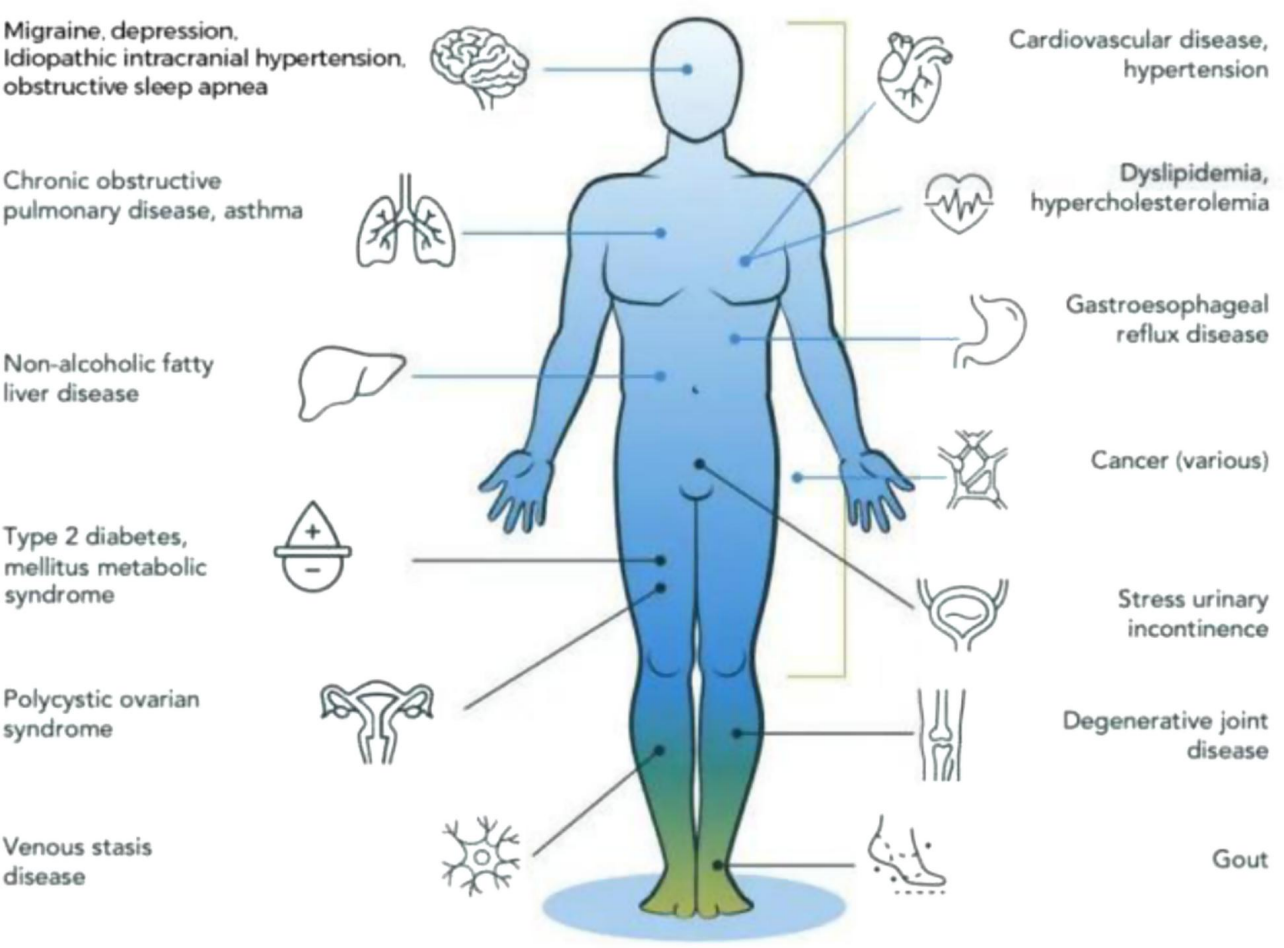
Effects of aging on major organs and tissues throughout the body.

**FIGURE 2:**
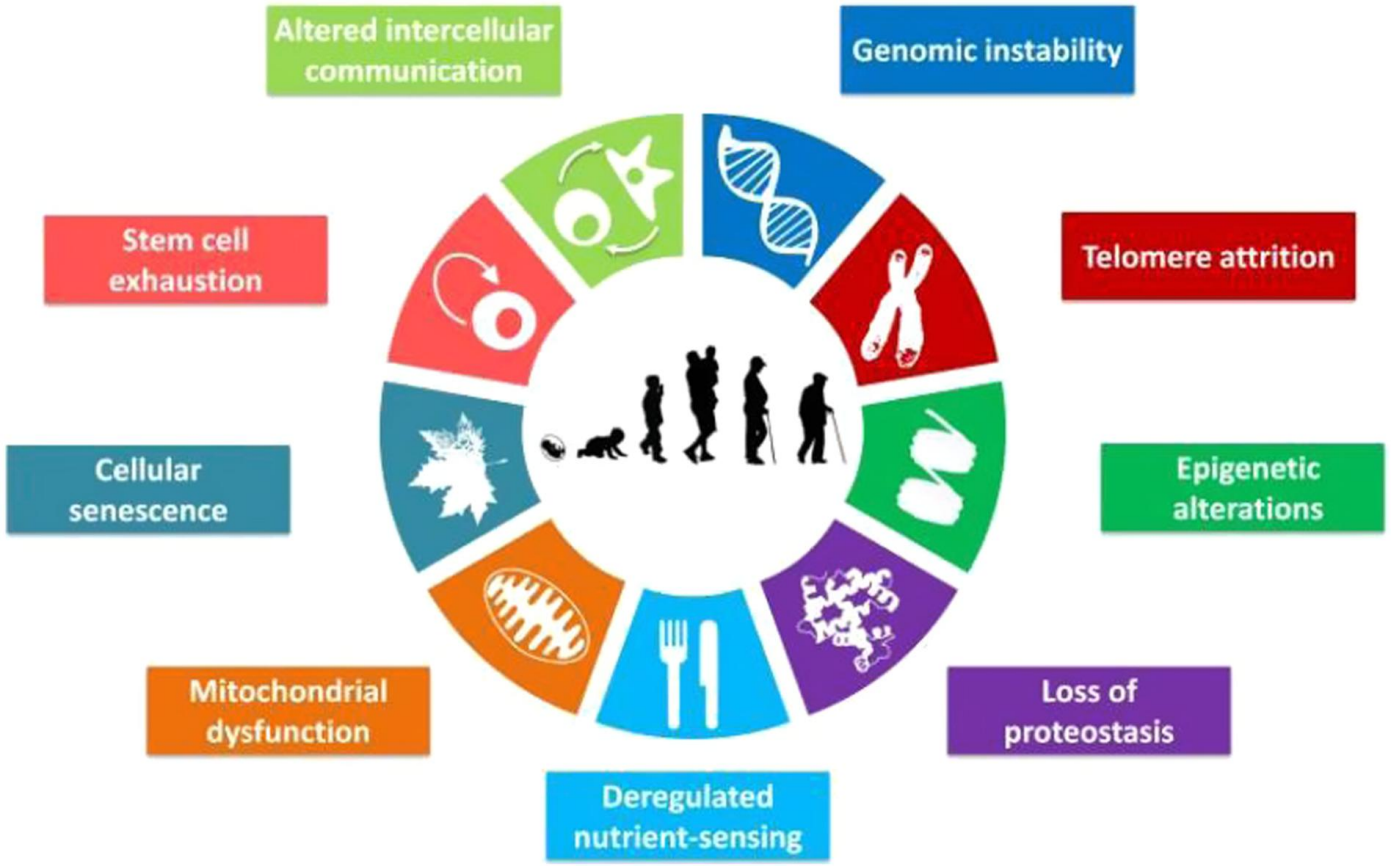
Characteristics of aging: cellular senescence, mitochondrial dysfunction, deregulated nutrient‐sensing, loss of proteostasis, epigenetic alterations, telomere attrition, genomic instability, altered intercellular communication and stem cell exhaustion. Reproduced with permission.^9^ Copyright 2013, Elsevier.

Previously, aging was thought to be a degenerative process, usually accompanied by the degeneration of organ structure and function^8^ (Figure 1). With the in‐depth research of cell biology in recent years, aging is considered to be a complex process, which can be summarized as cellular senescence, genomic instability, telomere attrition, epigenetic alterations, loss of proteostasis, nutrient sensing deregulation, mitochondrial dysfunction, stem cell exhaustion, and altered intercellular communication (Figure 2).^10^ Among these hallmarks, cellular senescence is considered to be the major driving factor of aging after injury. By analyzing the whole process of damage repair, scholars have found that the presence of senescent cells early in damage repair has been shown to be beneficial for damage repair. They can play a positive role by releasing specific signaling molecules to promote cell proliferation and new tissue formation. However, sustained accumulation of senescent cells will lead to chronic inflammation, altered cell microenvironment, and impaired stem cell repair function. Together, these negative effects severely weaken the tissue's ability to repair and regenerate.^11^ Based on this, clearing senescent cells or disrupting their effects on adjacent cells, while establishing a pro‐neogenesis microenvironment, serves the purpose of alleviating aging and promoting tissue regeneration. This will be a new therapeutic strategy for tissue engineering and regenerative medicine.

In summary, in this review, we believe that with the in‐depth research on aging and regeneration after tissue injury, it marks a turning point of an era. Research on inhibiting the accumulation of senescent cells to promote tissue regeneration in regenerative medicine has considerable potential.

## 2 MARKERS AND MICROENVIRONMENT OF SENESCENT CELLS

### 2.1 Characteristics of cell senescence

**FIGURE 3:**
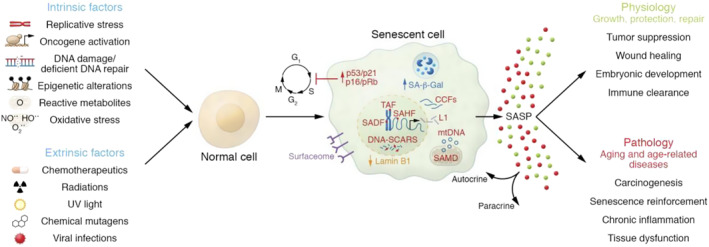
Various stress stimuli induce cellular senescence, with senescent cells exhibiting pleiotropic effects in physiology and pathology. Reproduced with permission.^12^ Copyright 2022, American Society for Clinical Investigation.

Cell senescence is a complex biological process, whose main features include loss of cellular proliferation capacity, changes in morphology and phenotype, production of specific secretory substances (such as the senescence‐associated secretory phenotype [SASP]), stable activation of cell cycle checkpoint factors (such as p53 and p16INK4a), and reorganization of chromatin structure (Figure 3). These features collectively impact cell function and intercellular interactions, which can lead to a decline in tissue function and manifestation of aging symptoms. Compared with ordinary cells, senescent cells have a prominent appearance of hypertrophy and flatness, which is mainly due to the accumulation of lysosomal particles in the cytoplasm.^13^ Senescence related β‐Galactosidase (SA‐β‐Gal) is the main method used to label and detect senescent cells, which refers to the catalytic bluing of senescent cells in the presence of X‐galactose under pH 6.0 conditions by expanding the amount and activity of SA‐β‐Gal in lysosomes.

Moreover, senescent cells have specific markers. The expression of p16^*INK4a*^ and p21^*CIP1*^ is increased in senescent cells. P16^INK4a^, p21^CIP1^ and p53 are cyclin‐dependent kinase inhibitors and tumor suppressors, which regulate cell‐programmed apoptosis in the G_1_ phase of cells in a coordinated and/or independent manner.^14^, ^15^, ^16^ Senescent cells are in a permanent growth arrest state^17^ in which the activation of p16^INK4a/Rb^ and/or p53/p21^CIP1^ tumor inhibition pathways inhibit cell‐programmed apoptosis. In addition, in the process of cell senescence, the nuclear membrane is destroyed, and lamin B1 is significantly reduced, which is another important marker of senescent cells.^18^ It is worth noting that senescent cells are closely related to DNA damage. DNA fragments with chromatin changes (DNA‐SCARS) and senescence‐related heterochromatin lesions in senescent cells are significantly enhanced.^19^, ^20^

### 2.2 Microenvironment of senescent cells

**FIGURE 4:**
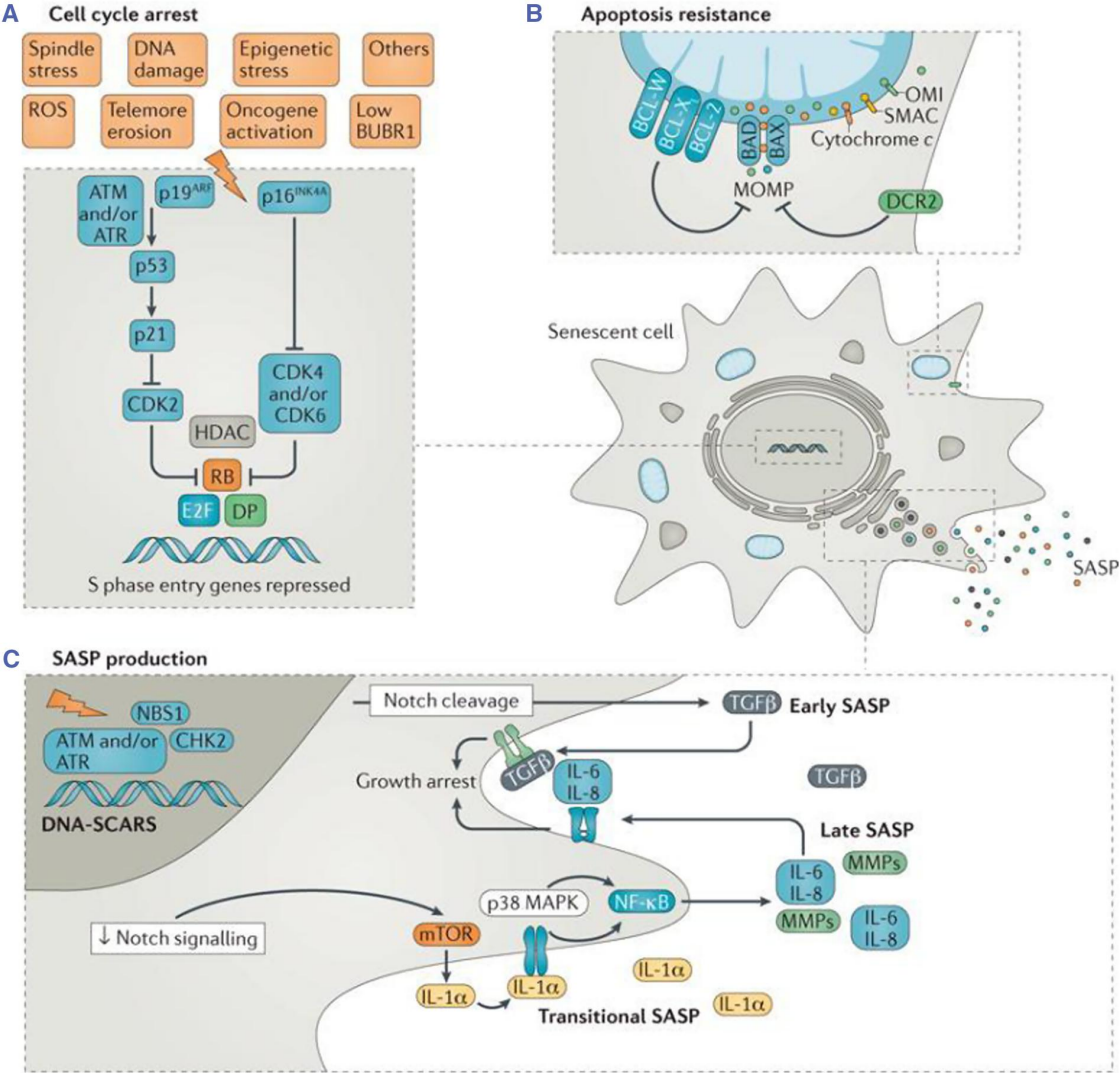
Senescent cells exhibit three key hallmarks: permanent cell cycle arrest, resistance to apoptotic signaling, and secretion of the senescence‐associated secretory phenotype (SASP). Reproduced with permission.^23^ Copyright 2017, Springer Nature.

The harmful effects of the microenvironment in senescent cell‐related diseases may be mediated by increased expression of SASP.^21^, ^22^ Generally speaking, senescent cells are thought to have three characteristics: cell proliferation arrest, apoptosis resistance and complex SASP (Figure 4). Senescent cells secrete various bioactive factors, including inflammatory cytokines, chemokines, growth factors, matrix metalloproteinases, lipids, nucleotides, extracellular vesicles and soluble factors, which is called SASP.

SASP produces abundant secretions and interacts with the external environment, which affects the formation of senescence and inflammation promoting microenvironment around its cells. Firstly, injecting a small number of pre‐senescence adipocytes led to extensive physical dysfunction in young mice.^21^ SASP acts in both proximal and distal ways to induce secondary senescence, thereby enhancing cell senescence.^24^ Secondly, SASP also helps to maintain and enhance the pro‐inflammatory microenvironment that allows chronic inflammation to occur with gradual intensification in the absence of pathogenic processes.^24^, ^25^ Inflammatory response in the early stage of injury can promote the proliferation of different cell groups to restore tissue damage. However, with the accumulation of senescent cells, the secretion of SASP increases, and the increase in inflammation related immune infiltration leads to the loss of tissue regenerative capacity and the increasing risk of disease with the accumulation of inflammatory factors.^26^ In conclusion, the accumulation of SASP secreted by senescent cells in the late stage of the tissue repair process can damage tissue remodeling.

## 3 EFFECT OF THE ACCUMULATION OF SENESCENT CELLS ON TISSUE REGENERATION

Many animals have the capacity to regenerate. By observing renewable organisms, researchers found that preventing the accumulation of senescent cells plays a crucial role.^26^ Salamanders are regarded as champion regenerators because they have a surprising regenerative ability: they can regenerate limbs, tails, hearts and some other organs, and they can maintain this ability even in adulthood. Senescent cells do not accumulate in tissues after several amputations. Macrophages clear senescent cells in the recovery area during regeneration, while not interfering with the proliferation of normal cells.^27^ Macrophage clearance of senescent cells effectively prevents senescent cell accumulation, which may be the key to the capacity of adult salamanders to have complete tissue regeneration.

**FIGURE 5:**
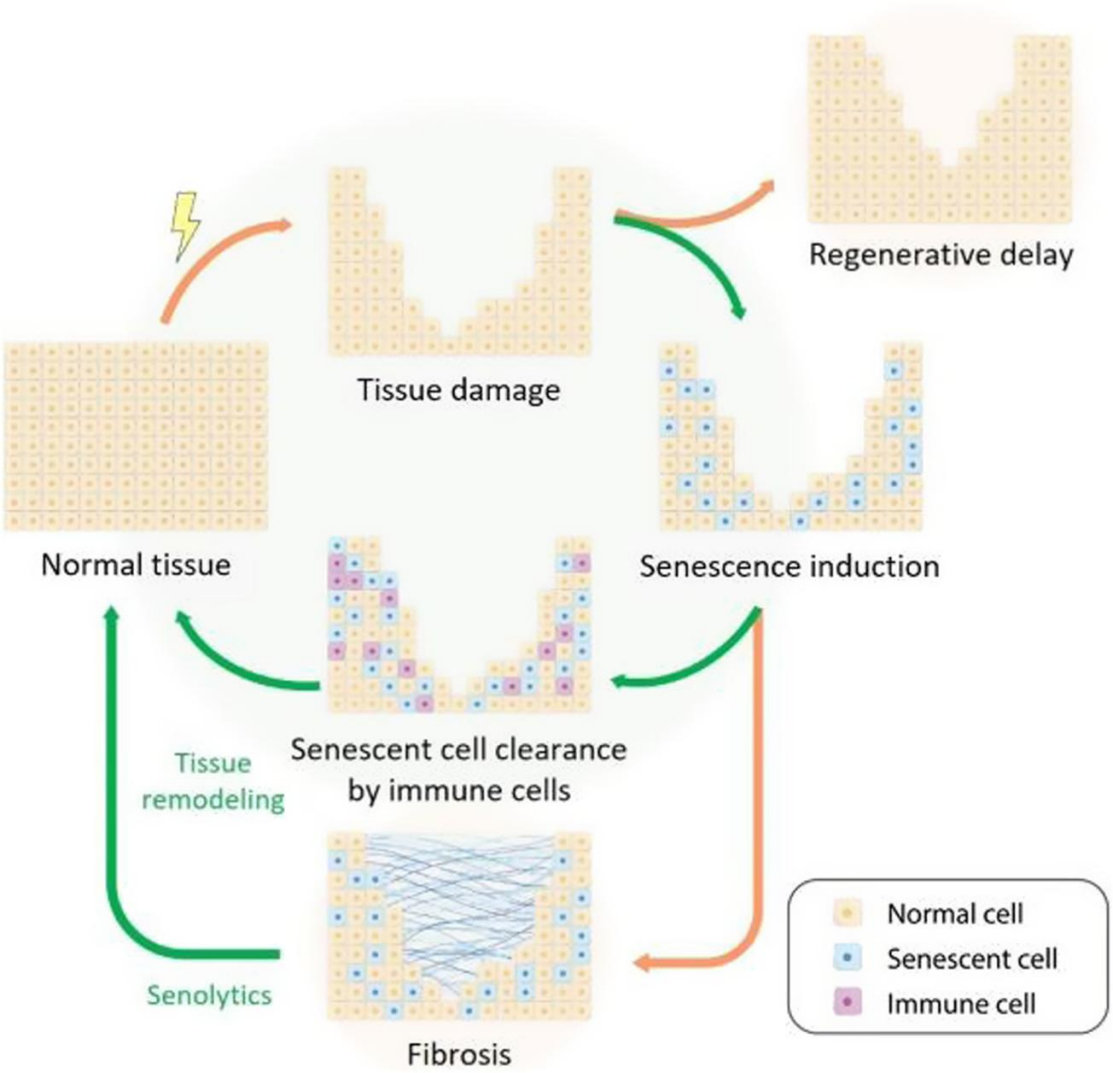
Role of senescent cells in tissue regeneration. Tissue damage triggers the formation of senescent cells, which in the injured area, promote repair through induction of dedifferentiation and proliferation, ultimately being cleared by recruited and activated immune cells. The accumulation of senescent cells beyond the capacity of immune cell clearance leads to their persistence in damaged tissues, inhibiting tissue repair and regeneration, eventually leading to tissue fibrosis. Interventions to prevent the accumulation of senescent cells aid in promoting tissue regeneration and remodeling. Reproduced with permission.^28^ Copyright 2021, Elsevier.

However, mammals do not have the capacity to completely recover lost limbs, but they only have the capacity to repair the partially lost tissue after injury. Senescent cells also play an important role in the process of tissue repair (Figure 5). Firstly, senescent cells are essential to skin repair after injury. When the mice skin tissue were damaged, there was a brief increase in senescent cells, and early senescent fibroblasts and endothelial cells emerged in the skin wounds.^29^ They participate in the formation of granulation tissue in the process of skin healing by secreting a SASP, platelet‐derived growth factor AA, and induce fibroblast differentiation to accelerate wound closure.^30^ It seems that the existence of senescent cells is beneficial for skin repair. However, long‐term overexposure to SASP can also promote the formation of more senescent cells in the keratin to resist regeneration stimulation and promote regeneration inhibition.^31^

Another important tissue for cell senescence during regeneration is muscle. In the muscle injury model of mice, skeletal muscle injury leads to the proliferation of fibrous adipose progenitor cells (FAPs), and gradual muscle stem cell differentiation. FAPs at the injury site have characteristic markers of senescence, such as the positive SA‐β‐Gal, the increase in H2AX and the rise of mRNA levels of Cdkn2a and Trp53 in cells (encoding p16 and p53, respectively). After the repair of muscle tissue is completed, the senescent FAPs enter the apoptosis process and are cleared by macrophages, returning to the level before injury.^32^ In terms of joints, the expression of p16Ink4a in synovium and cartilage surface cells increased gradually after joint injury and decreased with injury repair, but remained higher than the basic level. Eliminating senescent cells through drugs can reduce articular cartilage erosion, pain and inflammatory markers. At the same time, the elimination of specific senescent cells can significantly reduce the occurrence of osteoarthritis and help to create an environment conducive to cartilage formation.^33^

The cardiac injury in mice shows significant accumulation of senescent cells. After apical resection, myocardial cells at the ischemic site showed positive SA‐β‐gal, and expressions of p16, p21, p53 and other aging markers.^34^ Meanwhile, some SASP markers such as MMP2 and MMP9 were also detected.^35^ Transplanting different progenitor cell (CPC) hearts after myocardial infarction in mice showed that the normal CPC transplanted mice showed improved cardiac function, while the senescent CPC transplanted mice did not recover, and even suffered from impaired cardiac function. This may indicate that the accumulation of senescent cells reduces the regenerative capacity of CPC and prevents them from differentiating into cardiomyocytes. But when removing these senescent CPCs, the proliferation and differentiation of resident CPCs into cardiomyocytes can be improved, and the formation of fibrosis may be reduced.^36^

Similarly, after spinal cord injury in mice, nerve cells showed obvious characteristics of senescent cells, including the positive SA‐β‐Gal staining, the expressions of cell cycle inhibitors (p21 or p16) and DNA damage markers (γ‐H2AX), the loss of proliferation markers (BrdU), and senescent cells' accumulation at the lesion edge in this area increased over time.^37^ It was found that the motor function of mice was significantly improved after the targeted elimination of senescent cells in this area. Meanwhile, the myelin sheath was preserved after injury, nerve axons were regenerated, and the formation of nerve tissue fibrosis and scar was reduced. Therefore, it can be considered that the accumulation of senescent cells in spinal cord injury mice is harmful and seriously affects the recovery of neural function.

**FIGURE 6:**
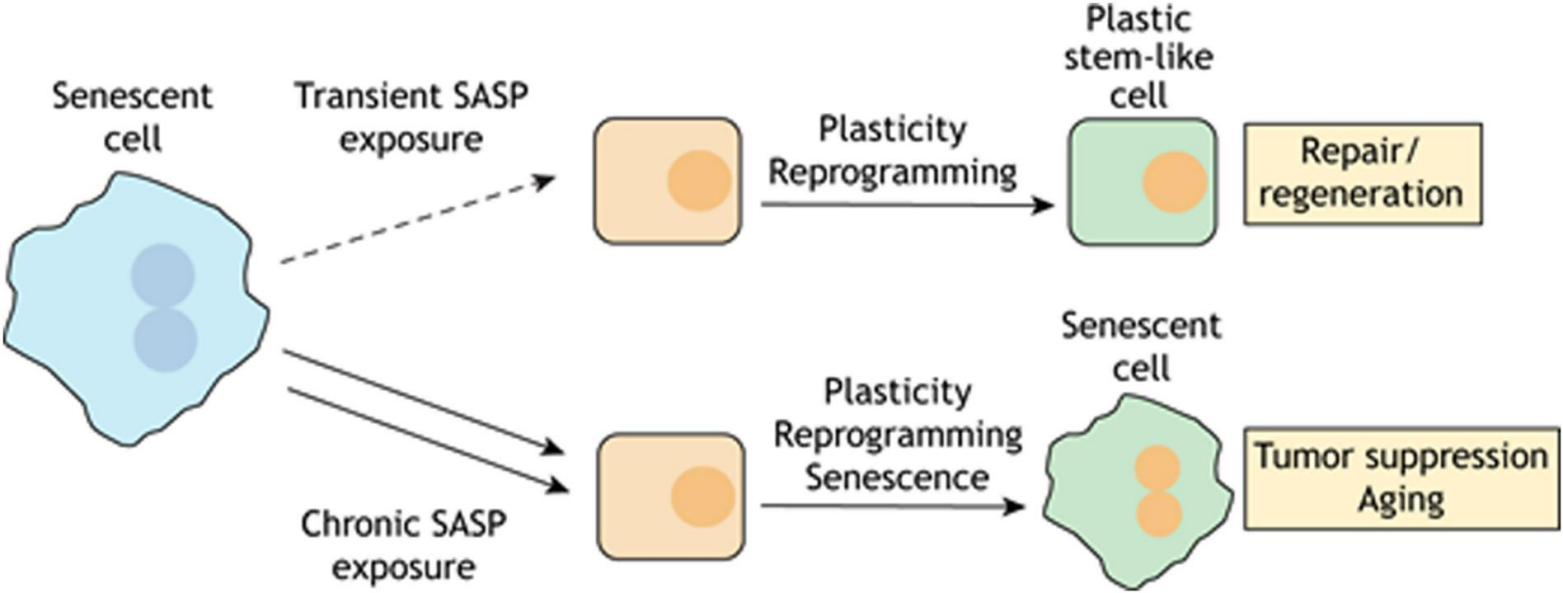
Brief exposure to SASP promotes tissue regenerative capacity, ultimately benefiting tissue repair and regeneration. However, prolonged exposure to SASP activates intrinsic senescence arrest, leading to the ultimate suppression of regeneration and/or senescence. Reproduced with permission.^28^ Copyright 2021, Elsevier. SASP, senescence‐associated secretory phenotype.

Therefore, understanding the dynamics and roles of senescent cells after injury is important for developing effective therapeutic strategies to accelerate tissue repair and regeneration (Figure 6).

## 4 INHIBITION OF SENESCENCE CELL ACCUMULATION AFTER TISSUE INJURY

### 4.1 Direct targeting to eliminate senescent cells reduces the burden of tissue regeneration and promotes tissue regeneration

Based on the above theory, reducing the accumulation of senescent cells can promote tissue regeneration, and eliminating senescent cells can directly solve the root cause of the problem. Drugs that can recognize and selectively kill senescent cells are called senolytics. Most senolytics are small molecule compounds.^38^, ^39^ The mechanism of these drugs is to recognize the up‐regulated anti‐apoptosis system in senescent cells, for example, the BCL‐2 protein family (BCL‐2, BCL‐XL and BCL‐W) members bind and functionally neutralize apoptosis.^40^ Apoptosis promoting BCL‐2 protein activates BAX and BCL‐2 homologous antagonist/killer (BAK) protein to trigger mitochondrial outer membrane permeabilization, resulting in the release of cytochrome c to drive programmed cell death.^41^ ABT‐737 and ABT‐263, as representatives, combine BCL‐2, BCL‐XL and BCL‐W inhibitory grooves, which counteract the anti‐apoptosis function of senescent cells and initiate apoptosis of senescent cells.^42^ As an earlier generation of senolytics, ABT‐737 has been shown to induce programmed apoptosis of dermal cells using p19^ARF^, the target of lung epithelial senescence cells.^43^ Similarly, ABT‐263 has been proven to eliminate the senescence foam cells in atherosclerotic lesions and block the progression of senescence cell‐dependent atherosclerosis.^37^ ABT‐263 has also been proven to eliminate senescence nerve cells after spinal cord injury, and ultimately promote the repair of nerve tissue and function.^21^ The combination of the tyrosine kinase inhibitor dasatinib and the flavonoid quercetin has been proven to eliminate senescent cells in vivo.^44^, ^45^ The combination of dasatinib targeting receptor/tyrosine kinase SCAP, quercetin targeting BCL‐2/BCL‐XL, PI3K/AKT and p53/p21/serpine SCAP, D + Q induced apoptosis in two senescent cell types. It is encoursenescence that many new anti‐senescence drugs have been tested in mice and human cells or tissues. For example, piperlongumine has recently proven to have a good effect on clearing senescent cells in vitro. Researchers are currently developing some polypeptide anti‐senescence drugs. FOXO4‐related peptides use the strategy of targeting SCAPs to inhibit the PI3K/AKT/p53/p21/serpine SCAP pathway to achieve an anti‐senescence activity.

**FIGURE 7:**
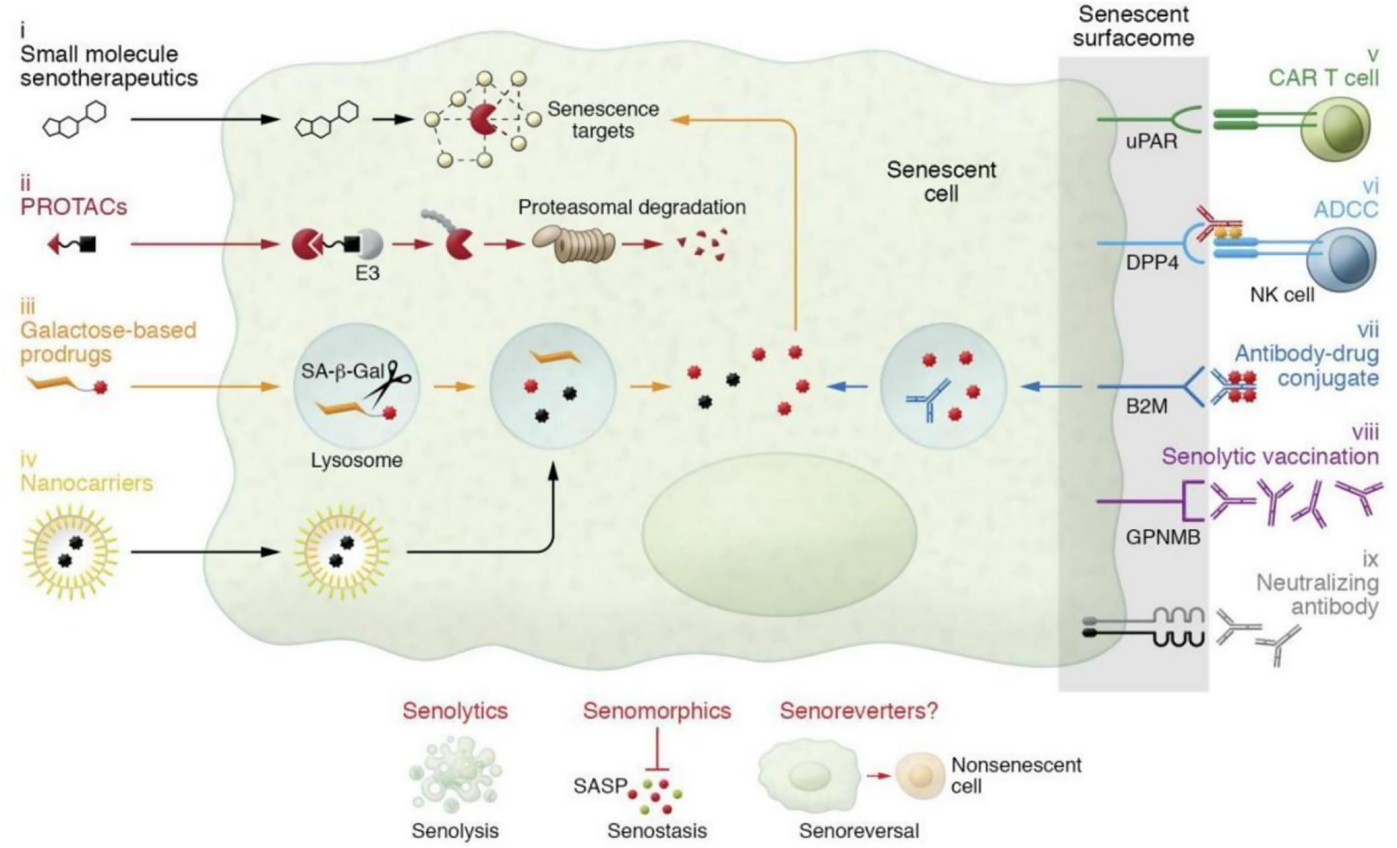
Current therapeutic strategies targeting senescent cells: senolytics and SASP inhibitors. Both intracellular senescence‐associated pathways and extracellular membrane proteins upregulated on the surface of senescent cells, termed the senescent surfaceome, can be exploited for therapeutic as well as diagnostic purposes. Current senotherapeutic strategies targeting senescent cells include conventional senotherapeutics, prodrugs, protein degraders, nanocarriers, and immunotherapies. Reproduced with permission.^12^ Copyright 2022, American Society for Clinical Investigation. SASP, senescence‐associated secretory phenotype.

In conclusion, some of the most promising anti‐senescence drugs have been transferred to clinical applications through preclinical research (Figure 7).

### 4.2 Regulated inflammatory and senescent microenvironment

The microenvironment can be improved by inhibiting the secretion of SASP in senescent cells. Many drugs have been found to have the capacity to regulate the secretion of SASP. Rusotinib is a JAK1/2 inhibitor which can reduce the secretion of granulocyte‐macrophage colony‐stimulating factor, granulocyte colony‐stimulating factor, CXCL1, IL‐6, IL‐8 and several other inflammatory SASP proteins in senescent cells by targeting the JAK/STAT pathway.^46^ Metformin is a type of anti‐diabetic drug, which is also recognized as an anti‐senescence drug. Its main mechanism is to inhibit IKKα/β and IκB phosphorylates and prevent p65 (RelA) nuclear translocation to weaken the secretion of SASP.^43^ Rapamycin is a selective inhibitor of mTOR complex 1, mainly by reducing IL‐1α. The efficiency of mRNA translation leads to the weakening of IL‐1R1 signal and the down‐regulation of NF‐κB controls the secretion of SASP.

MicroRNAs can bind with mRNAs to block the expression of protein coding genes. The control of SASP by microRNA usually involves the regulation of nuclear factor kB. In the senescence fibroblast model, miRNA146a/b has been proven to negatively regulate the secretion of IL‐6 and IL‐8 by controlling IL‐1 receptor related kinase 1.^47^ Extracellular microRNAs in body fluids may interact with Toll‐like receptors, thereby interfering with the production of many SASP factors. In human macrophages, miR‐21 interacts functionally with TLR8 in vivo through NF‐κB activation, which leads to the secretion of cytokines TNF‐α and IL‐6.^48^ MiR‐199a controls IKKβ expression of NF‐κB in ovarian cancer cells.^49^ Similarly, miR‐155 controls IKKβ and IKKε expression. NF‐κB inhibits inflammation related to SASP secretion and senescence,^50^ creating a microenvironment suitable for tissue regeneration.

## 5 INHIBITING CELLULAR SENESCENCE COMBINED WITH PRO‐REGENERATIVE BIOMATERIALS

The exploration of pro‐regenerative biomaterials plays a crucial role in regenerative medicine. These biomaterials, including biocompatible materials, bioactive materials, responsive materials and tissue engineering scaffolds, provide necessary environments for cell growth and tissue repair.^51^ They can mimic the internal biological environment of the human body, support cell growth, and even stimulate or guide cell behaviors. The design and fabrication of these materials need to consider not only the interactions with cells, biomolecules and in vivo environments, but also adjust their structures and properties according to specific tissue types (such as bone, cartilage or skin). By thoroughly investigating the interactions between these materials and cells, we can develop more effective strategies to maximize the formation of a pro‐regenerative microenvironment in damaged tissues. However, the exploration in the design of biomaterials that simultaneously inhibit cellular senescence and promote cell regeneration remains quite limited. Nonetheless, in the early stages of scholarly investigation, the potential for future clinical applications of this strategy is still evident.

**FIGURE 8:**
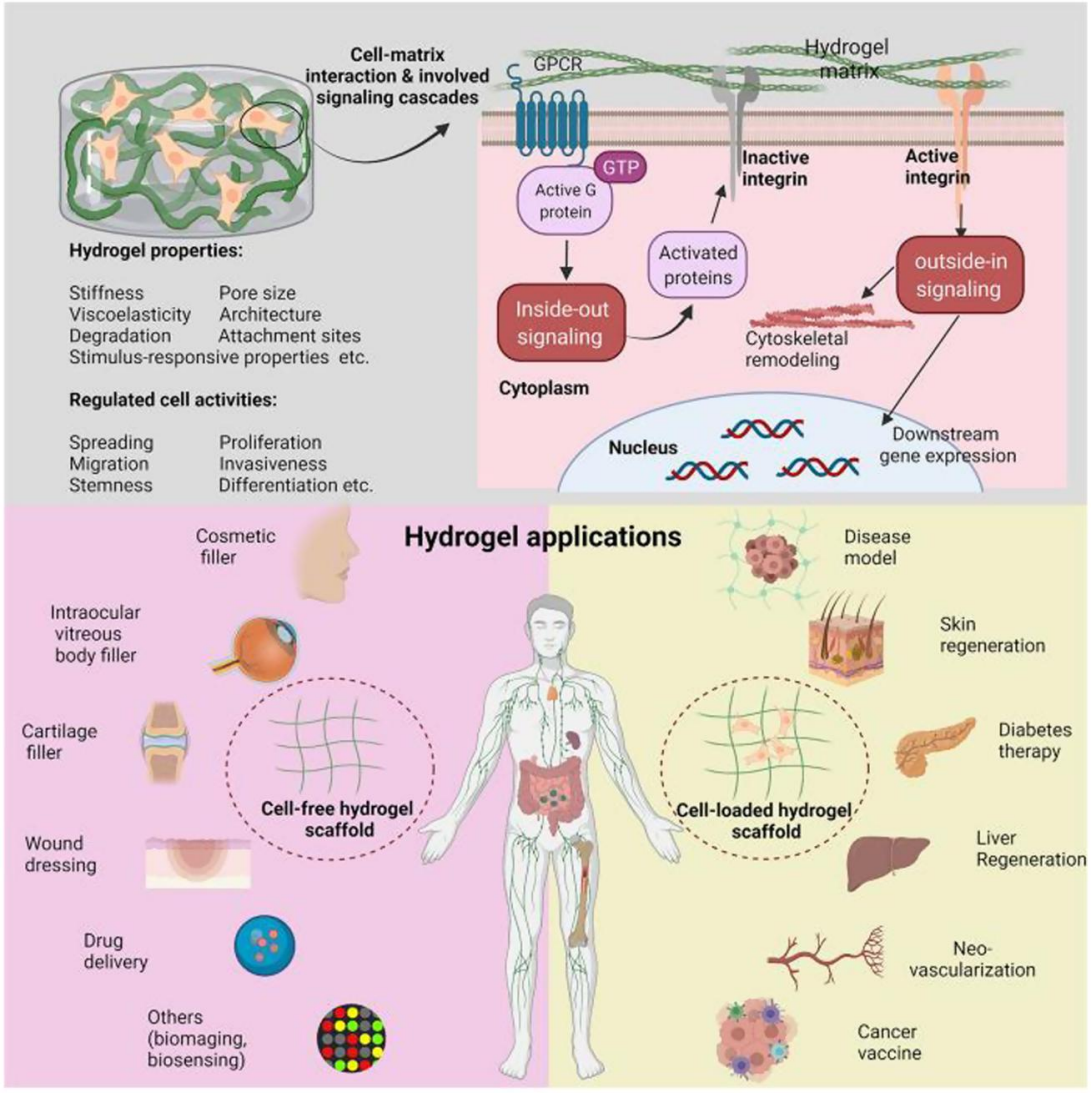
Advances in hydrogels in regenerative medicine.

Hydrogels play an important role in promoting cell neogenesis due to their good biocompatibility, tunability, and capability as carriers for drugs or growth factors (Figure 8). They provide an ideal 3D microenvironment for cell growth and can serve as tissue engineering scaffolds to facilitate cell localization, growth and differentiation.^52^ Moreover, by encapsulating drugs or growth factors in hydrogels, sustained and localized release of these bioactive substances can be achieved, which further promotes cell neogenesis, tissue repair and regeneration.^53^ Researchers have explored the encapsulation of the anti‐aging agent quercetin within hydrogels, which can selectively release quercetin in a responsive manner to target the formation of senescent cells. The quercetin‐loaded hydrogel effectively cleared local senescent cells and reduced the secretion of MMP in bone. By eliminating local aging cells, the hydrogel significantly accelerated the repair of bone defects in aged rats.^54^ Furthermore, some scholars have proposed a hydrogel‐based miRNA delivery strategy for treating osteoarthritis. This approach involves mitigating the formation of senescent chondrocytes by regulating the aging‐related miR‐29b‐5p. Simultaneously, the hydrogel can be functionalized by incorporating stem cell homing peptide SKPPGTSS, recruiting endogenous synovial stem cells to facilitate regeneration. Ultimately, sustained miR‐29b‐5p delivery coupled with the recruitment and subsequent differentiation of synovial stem cells into chondrocytes, leads to successful cartilage repair and chondrocyte regeneration.^55^ Therefore, these unique properties of hydrogels provide powerful tools for cell neogenesis and regenerative medicine.

**FIGURE 9:**
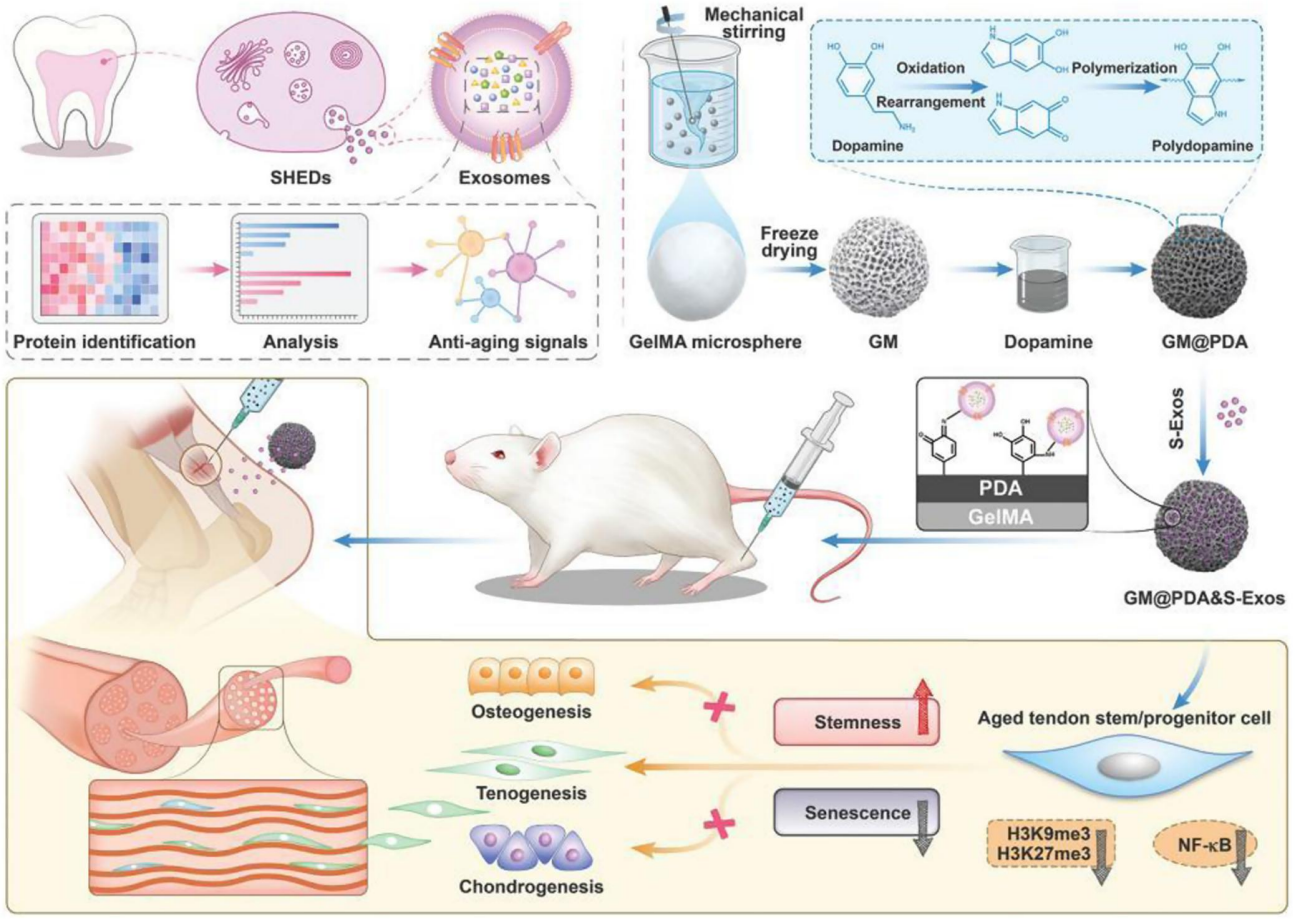
Young exosome bio‐Nanoparticles restore aging‐Impaired tendon stem/progenitor cell function and reparative capacity.

Microspheres prepared from hydrogels using microfluidic techniques are also important in facilitating cell neogenesis.^56^ Microspheres can serve as cell carriers that encapsulate and protect cells for release at specific times and locations, such as for transplanting stem cells or other therapeutic cells to treat damaged tissues (Figure 9).^57^ In addition, encapsulating drugs or growth factors in hydrogel microspheres allows their release at specific times and locations, which can improve therapeutic efficacy and reduce side effects. Hydrogel microspheres can also mimic the natural microenvironment of cells to support cell growth and differentiation, and construct 3D tissue engineering scaffolds to provide space for cell growth and tissue formation. Research has indicated that hydrogel microspheres loaded with anti‐aging exosomes can effectively aid in tendon reconstruction. Combining exosomes from young human exfoliated deciduous tooth stem cells with hydrogel microspheres has been shown to alleviate the aging phenotype of tendon stem cells and maintain their tendon regeneration capacity. Mechanistically, these hydrogel microspheres can modulate histone methylation and inhibit nuclear factor‐κB, thus reversing the aging of tendon stem cells. It is noteworthy that local delivery of young exosome microspheres imparts anti‐aging phenotypes to cells, including reducing aging cells and diminishing ectopic bone formation, ultimately rescuing the endogenous tendon regeneration and repair ability in aged rats (Figure 9).^58^ Overall, hydrogel microsphere particles, as natural bioactive nanocarriers, hold significant translational and therapeutic potential for aging‐related diseases.

**FIGURE 10:**
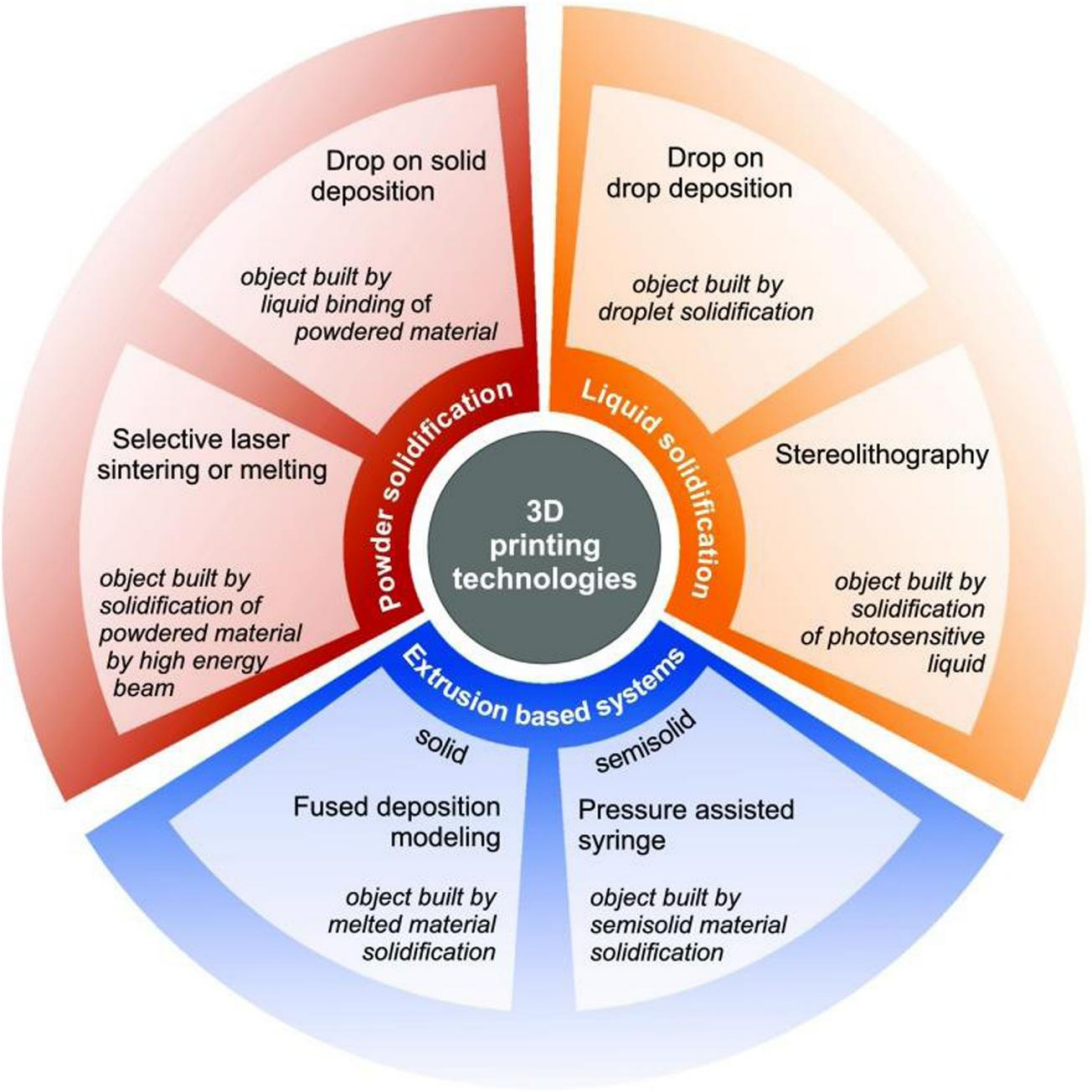
Applications of 3D Printing Technology in Medicine. 3D printing technology has been explored in the fields of tissue engineering, drug testing and screening, regenerative medicine and clinical disease research and has achieved many research results.

Benefiting from the precision and customizability of 3D printing technology, it has been gradually applied in regenerative medicine (Figure 10). First, 3D printing can precisely construct complex microscopic structures that mimic the native environments of cells, promoting cell adhesion, growth and differentiation. Second, 3D printing can be utilized to manufacture biomaterials of various shapes and sizes that can be customized according to patients' specific needs, thereby improving therapeutic adaptability.^59^ Moreover, 3D printing enables the fabrication of biomaterials carrying drugs or growth factors that can be released at specific times and locations to achieve fine modulation of the cell microenvironment and further promote cell neogenesis. Wu et al.^60^ utilized 3D printing technology to fabricate isoniazid sustained‐release implants and found that the drug concentration remained above the effective antibacterial level even after 30 days of in vitro release. The results of this study suggest that the produced implants are an ideal antibiotic delivery system, and this technology offers a reliable method for creating complex implants combined with drugs. Huang et al.^61^ developed implants containing levofloxacin for complex drug release profiles. Levofloxacin implants achieved a dual‐mode release profile with pulsatile and steady‐state release. In summary, 3D printing technology can manufacture drug implants with both complex micro and macrostructures in a single device, making it easier for prototyping and manufacturing. It offers distinct advantages over traditionally manufactured implants and provides a powerful tool for drug delivery and tissue engineering applications.

**FIGURE 11:**
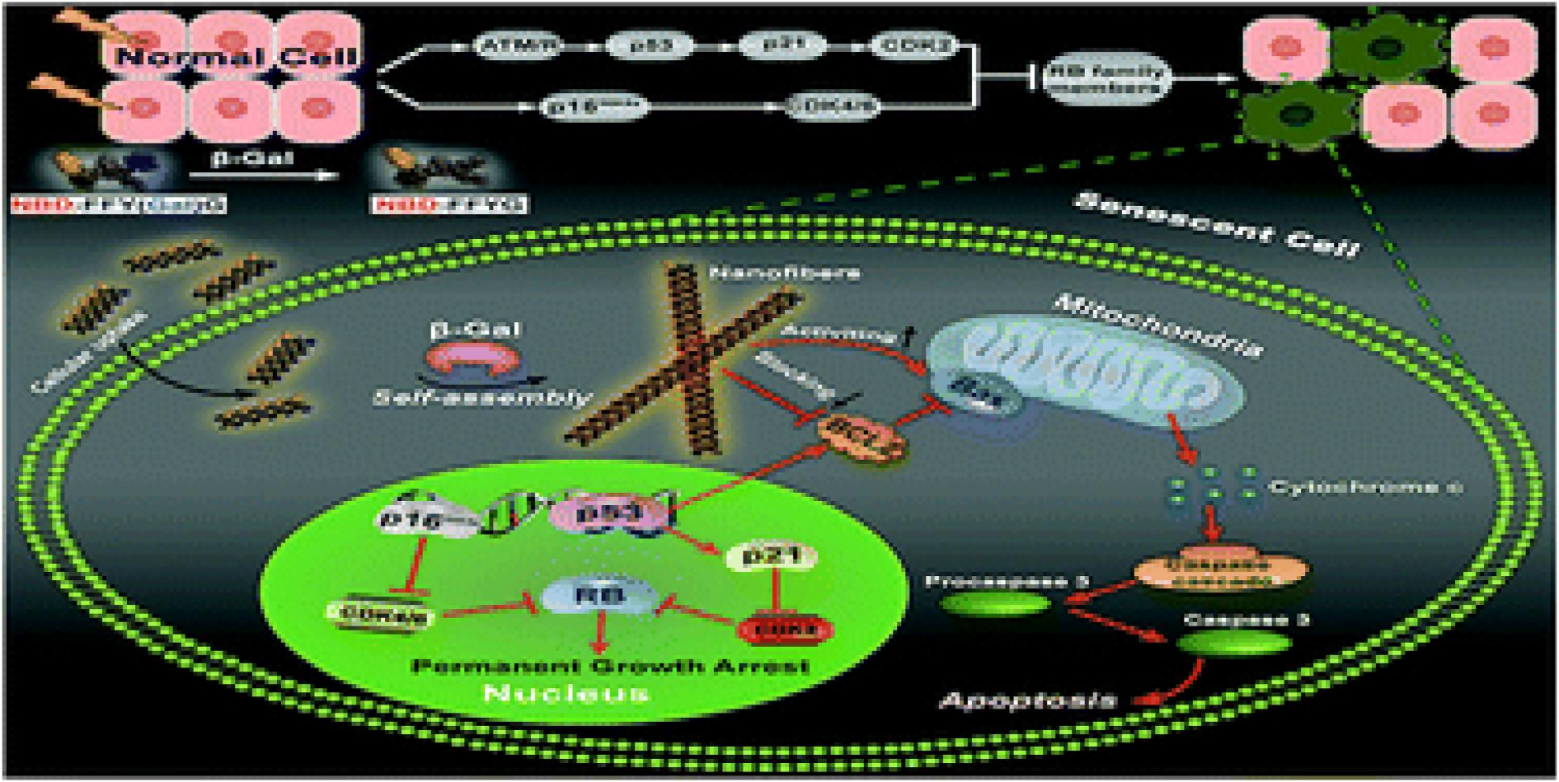
Beta‐galactosidase instructed supramolecular hydrogelation for selective dentification and removal of senescent cells.

Nanofibers can provide the following effects on neogenic cells owing to their unique structural features. Firstly, the micro‐structure of nanofibers can mimic native extracellular matrix, providing a biomimetic growth substrate facilitating cell adhesion, growth and differentiation.^62^ Second, due to the good surface properties and high specific surface area, nanofibers can be used as carriers for drugs or growth factors to achieve fine modulation of cell microenvironment, further promoting cell neogenesis. Moreover, nanofibers can also act as tissue engineering scaffolds to facilitate the repair of damaged tissues.^63^ Emanuela Bellu et al.^64^ employed specific molecules to prepare nanofibers to prevent skin aging caused by ultraviolet exposure, aiming to maintain its youthful phenotype. The preparation of nanofibers allowed for the organized delivery of anti‐aging Mediterranean plant extract, Helichrysum italicum. The study found that these nanofibers could resist the aging effects induced by ultraviolet radiation on adipose stem cells and dermal fibroblasts, thus preserving the youthful characteristics of these cells. It's worth noting that Xu et al.,^65^ inspired by the higher expression of β‐galactosidase (β‐Gal) in aging cells, designed a β‐Gal‐responsive precursor that selectively formed nanofibers through Enzyme‐Induced Self‐Assembly upon enzymatic action. This approach enables the detection and clearance of aging cells. This strategy has applications in the diagnosis and treatment of chronic diseases and holds significant value in tissue regeneration (Figure 11). In summary, these features endow nanofibers with broad application potentials in cell neogenesis and regenerative medicine.

In general, the above explorations involve not only designing and preparing new materials, but also combining them with cells, biomolecules and in vivo environments to maximize their functions. By thoroughly understanding the interactions between cells and materials, we can develop more effective strategies to promote tissue repair and regeneration.

## 6 CONCLUSION

In the field of regenerative medicine, inhibiting cellular senescence to promote cell regeneration is an emerging research direction. Combining senolytics and SASP inhibitors to clear senescent cells with emerging biomaterials that effectively promote tissue neogenesis may pioneer a potentially revolutionary strategy. The therapeutic strategy of inhibiting cellular senescence combined with pro‐regenerative biomaterials could bring significant benefits to numerous trauma patients. However, since this is still a relatively new therapeutic strategy, many challenges and barriers need to be addressed before its clinical application. But we believe that through continuous research and effort, the strategy of clearing senescent cells to promote cell regeneration will provide new research and application perspectives for regenerative medicine.

## AUTHOR CONTRIBUTIONS

The manuscript was written through the contributions of all authors. All authors have given approval to the final version of the manuscript.

## CONFLICT OF INTEREST STATEMENT

The authors declare that there are no competing interests.

## References

[1] L. Edgar , T. Pu , B. Porter , J. M. Aziz , C. La Pointe , A. Asthana , G. Orlando , Br. J. Surg. 2020, 107, 793. 32463143 10.1002/bjs.11686

[2] F. Berthiaume , T. J. Maguire , M. L. Yarmush , Annu. Rev. Chem. Biomol. Eng. 2011, 2, 403. 22432625 10.1146/annurev-chembioeng-061010-114257

[3] G. Orlando , S. V. Murphy , B. Bussolati , M. Clancy , P. Cravedi , G. Migliaccio , P. Murray , Transplantation 2019, 103, 237. 30028414 10.1097/TP.0000000000002370

[4] M. J. Yousefzadeh , R. R. Flores , Y. Zhu , Z. C. Schmiechen , R. W. Brooks , C. E. Trussoni , Y. Cui , L. Angelini , K. A. Lee , S. J. McGowan , A. L. Burrack , D. Wang , Q. Dong , A. Lu , T. Sano , R. D. O’Kelly , C. A. McGuckian , J. I. Kato , M. P. Bank , E. A. Wade , S. P. S. Pillai , J. Klug , W. C. Ladiges , C. E. Burd , S. E. Lewis , N. F. LaRusso , N. V. Vo , Y. Wang , E. E. Kelley , J. Huard , I. M. Stromnes , P. D. Robbins , L. J. Niedernhofer , Nature 2021, 594, 100. 33981041 10.1038/s41586-021-03547-7 PMC8684299

[5] A. Brunet , M. A. Goodell , T. A. Rando , Nat. Rev. Mol. Cell Biol. 2023, 24, 45. 35859206 10.1038/s41580-022-00510-w PMC9879573

[6] X. Hong , S. Campanario , I. Ramírez‐Pardo , M. Grima‐Terrén , J. Isern , P. Muñoz‐Cánoves , Ageing Res. Rev. 2022, 73, 101528. 34818593 10.1016/j.arr.2021.101528

[7] R. A. Avelar , J. G. Ortega , R. Tacutu , E. J. Tyler , D. Bennett , P. Binetti , A. Budovsky , K. Chatsirisupachai , E. Johnson , A. Murray , S. Shields , D. Tejada‐Martinez , D. Thornton , V. E. Fraifeld , C. L. Bishop , J. P. de Magalhães , Genome Biol. 2020, 21, 91. 32264951 10.1186/s13059-020-01990-9 PMC7333371

[8] B. K. Kennedy , S. L. Berger , A. Brunet , J. Campisi , A. M. Cuervo , E. S. Epel , C. Franceschi , G. J. Lithgow , R. I. Morimoto , J. E. Pessin , T. A. Rando , A. Richardson , E. E. Schadt , T. Wyss‐Coray , F. Sierra , Cell 2014, 159, 709. 25417146 10.1016/j.cell.2014.10.039 PMC4852871

[9] C. Lopez‐Otin , M. A. Blasco , L. Partridge , M. Serrano , G. Kroemer , Cell 2013, 153, 1194. 23746838 10.1016/j.cell.2013.05.039 PMC3836174

[10] C. Lopez‐Otin , M. A. Blasco , L. Partridge , M. Serrano , G. Kroemer , Cell 2023, 186, 243. 36599349 10.1016/j.cell.2022.11.001

[11] S. J. Morrison , A. C. Spradling , Cell 2008, 132, 598. 18295578 10.1016/j.cell.2008.01.038 PMC4505728

[12] L. Zhang , L. E. Pitcher , M. J. Yousefzadeh , L. J. Niedernhofer , P. D. Robbins , Y. Zhu , J. Clin. Invest. 2022, 132, e158450. 35912854 10.1172/JCI158450 PMC9337830

[13] B. Y. Lee , J. A. Han , J. S. Im , A. Morrone , K. Johung , E. C. Goodwin , W. J. Kleijer , D. DiMaio , E. S. Hwang , Aging Cell 2006, 5, 187. 16626397 10.1111/j.1474-9726.2006.00199.x

[14] J. W. Shay , O. M. Pereira‐Smith , W. E. Wright , Exp. Cell Res. 1991, 196, 33. 1652450 10.1016/0014-4827(91)90453-2

[15] C. M. Beausejour , A. Krtolica , F. Galimi , M. Narita , S. W. Lowe , P. Yaswen , J. Campisi , EMBO J. 2003, 22, 4212. 12912919 10.1093/emboj/cdg417 PMC175806

[16] M. Serrano , G. J. Hannon , D. Beach , Nature 1993, 366, 704. 8259215 10.1038/366704a0

[17] B. G. Childs , M. Durik , D. J. Baker , J. M. van Deursen , Nat. Med. 2015, 21, 1424. 26646499 10.1038/nm.4000 PMC4748967

[18] I. Matias , L. P. Diniz , I. V. Damico , A. Araujo , L. Neves , G. Vargas , R. Leite , C. K. Suemoto , R. Nitrini , W. Jacob‐Filho , L. T. Grinberg , E. M. Hol , J. Middeldorp , F. Gomes , Aging Cell 2022, 21, e13521. 34894056 10.1111/acel.13521 PMC8761005

[19] F. Rodier , D. P. Munoz , R. Teachenor , V. Chu , O. Le , D. Bhaumik , J. P. Coppe , E. Campeau , C. M. Beausejour , S. H. Kim , A. R. Davalos , J. Campisi , J. Cell Sci. 2011, 124, 68. 21118958 10.1242/jcs.071340 PMC3001408

[20] R. Zhang , P. D. Adams , Cell Cycle 2007, 6, 784. 17377503 10.4161/cc.6.7.4079

[21] J. P. Coppe , P. Y. Desprez , A. Krtolica , J. Campisi , Annu. Rev. Pathol. Mech. Dis. 2010, 5, 99. 10.1146/annurev-pathol-121808-102144 PMC4166495 20078217

[22] S. Lopes‐Paciencia , E. Saint‐Germain , M. C. Rowell , A. F. Ruiz , P. Kalegari , G. Ferbeyre , Cytokine 2019, 117, 15. 30776684 10.1016/j.cyto.2019.01.013

[23] B. G. Childs , M. Gluscevic , D. J. Baker , R. M. Laberge , D. Marquess , J. Dananberg , J. M. van Deursen , Nat. Rev. Drug Discovery 2017, 16, 718. 28729727 10.1038/nrd.2017.116 PMC5942225

[24] M. Xu , T. Pirtskhalava , J. N. Farr , B. M. Weigand , A. K. Palmer , M. M. Weivoda , C. L. Inman , M. B. Ogrodnik , C. M. Hachfeld , D. G. Fraser , J. L. Onken , K. O. Johnson , G. C. Verzosa , L. G. P. Langhi , M. Weigl , N. Giorgadze , N. K. LeBrasseur , J. D. Miller , D. Jurk , R. J. Singh , D. B. Allison , K. Ejima , G. B. Hubbard , Y. Ikeno , H. Cubro , V. D. Garovic , X. Hou , S. J. Weroha , P. D. Robbins , L. J. Niedernhofer , S. Khosla , T. Tchkonia , J. L. Kirkland , Nat. Med. 2018, 24, 1246. 29988130 10.1038/s41591-018-0092-9 PMC6082705

[25] K. Bandeen‐Roche , Q. Xue , L. Ferrucci , J. Walston , J. M. Guralnik , P. Chaves , S. L. Zeger , L. P. Fried , J. Gerontol. Ser. A 2006, 61, 262. 10.1093/gerona/61.3.262 16567375

[26] L. Ferrucci , E. Fabbri , Nat. Rev. Cardiol. 2018, 15, 505. 30065258 10.1038/s41569-018-0064-2 PMC6146930

[27] C. Franceschi , J. Campisi , J. Gerontol. Ser. A 2014, 69, S4. 10.1093/gerona/glu057 24833586

[28] L. Antelo‐Iglesias , P. Picallos‐Rabina , V. Estevez‐Souto , S. Da Silva‐Alvarez , M. Collado , Mech. Ageing Dev. 2021, 198, 111528. 34181964 10.1016/j.mad.2021.111528

[29] M. H. Yun , H. Davaapil , J. P. Brockes , eLife 2015, 4, e05505. 25942455 10.7554/eLife.05505 PMC4434796

[30] M. Storer , A. Mas , A. Robert‐Moreno , M. Pecoraro , M. C. Ortells , V. Di Giacomo , R. Yosef , N. Pilpel , V. Krizhanovsky , J. Sharpe , W. M. Keyes , Cell 2013, 155, 1119. 24238961 10.1016/j.cell.2013.10.041

[31] M. Demaria , N. Ohtani , S. A. Youssef , F. Rodier , W. Toussaint , J. R. Mitchell , R. M. Laberge , J. Vijg , H. Van Steeg , M. E. Dolle , J. H. Hoeijmakers , A. de Bruin , E. Hara , J. Campisi , Dev. Cell 2014, 31, 722. 25499914 10.1016/j.devcel.2014.11.012 PMC4349629

[32] B. Ritschka , M. Storer , A. Mas , F. Heinzmann , M. C. Ortells , J. P. Morton , O. J. Sansom , L. Zender , W. M. Keyes , Genes Dev. 2017, 31, 172. 28143833 10.1101/gad.290635.116 PMC5322731

[33] Y. Saito , T. S. Chikenji , T. Matsumura , M. Nakano , M. Fujimiya , Nat. Commun. 2020, 11, 889. 32060352 10.1038/s41467-020-14734-x PMC7021787

[34] O. H. Jeon , C. Kim , R. M. Laberge , M. Demaria , S. Rathod , A. P. Vasserot , J. W. Chung , D. H. Kim , Y. Poon , N. David , D. J. Baker , J. M. van Deursen , J. Campisi , J. H. Elisseeff , Nat. Med. 2017, 23, 775. 28436958 10.1038/nm.4324 PMC5785239

[35] R. Sarig , R. Rimmer , E. Bassat , L. Zhang , K. B. Umansky , D. Lendengolts , G. Perlmoter , K. Yaniv , E. Tzahor , Circulation 2019, 139, 2491. 31107623 10.1161/CIRCULATIONAHA.119.040125

[36] F. C. Lewis‐McDougall , P. J. Ruchaya , E. Domenjo‐Vila , T. Shin Teoh , L. Prata , B. J. Cottle , J. E. Clark , P. P. Punjabi , W. Awad , D. Torella , T. Tchkonia , J. L. Kirkland , G. M. Ellison‐Hughes , Aging Cell 2019, 18, e12931. 30854802 10.1111/acel.12931 PMC6516154

[37] D. Paramos‐de‐Carvalho , I. Martins , A. M. Cristóvão , A. F. Dias , D. Neves‐Silva , T. Pereira , D. Chapela , A. Farinho , A. Jacinto , L. Saúde , Cell Rep. 2021, 36, 109334. 34233184 10.1016/j.celrep.2021.109334

[38] J. Chang , Y. Wang , L. Shao , R. M. Laberge , M. Demaria , J. Campisi , K. Janakiraman , N. E. Sharpless , S. Ding , W. Feng , Y. Luo , X. Wang , N. Aykin‐Burns , K. Krager , U. Ponnappan , M. Hauer‐Jensen , A. Meng , D. Zhou , Nat. Med. 2016, 22, 78. 26657143 10.1038/nm.4010 PMC4762215

[39] R. Yosef , N. Pilpel , R. Tokarsky‐Amiel , A. Biran , Y. Ovadya , S. Cohen , E. Vadai , L. Dassa , E. Shahar , R. Condiotti , I. Ben‐Porath , V. Krizhanovsky , Nat. Commun. 2016, 7, 11190. 27048913 10.1038/ncomms11190 PMC4823827

[40] H. Fuhrmann‐Stroissnigg , Y. Ling , J. Zhao , S. J. McGowan , Y. Zhu , R. W. Brooks , D. Grassi , S. Q. Gregg , J. L. Stripay , A. Dorronsoro , L. Corbo , P. Tang , C. Bukata , N. Ring , M. Giacca , X. Li , T. Tchkonia , J. L. Kirkland , L. J. Niedernhofer , P. D. Robbins , Nat. Commun. 2017, 8, 422. 28871086 10.1038/s41467-017-00314-z PMC5583353

[41] M. Luna‐Vargas , J. E. Chipuk , Trends Cell Biol. 2016, 26, 906. 27498846 10.1016/j.tcb.2016.07.002 PMC5118054

[42] C. Tse , A. R. Shoemaker , J. Adickes , M. G. Anderson , J. Chen , S. Jin , E. F. Johnson , K. C. Marsh , M. J. Mitten , P. Nimmer , L. Roberts , S. K. Tahir , Y. Xiao , X. Yang , H. Zhang , S. Fesik , S. H. Rosenberg , S. W. Elmore , Cancer Res. 2008, 68, 3421. 18451170 10.1158/0008-5472.CAN-07-5836

[43] M. Xu , T. Tchkonia , H. Ding , M. Ogrodnik , E. R. Lubbers , T. Pirtskhalava , T. A. White , K. O. Johnson , M. B. Stout , V. Mezera , N. Giorgadze , M. D. Jensen , N. K. LeBrasseur , J. L. Kirkland , Proc. Natl. Acad. Sci. U. S. A. 2015, 112, E6301. 26578790 10.1073/pnas.1515386112 PMC4655580

[44] B. G. Childs , D. J. Baker , T. Wijshake , C. A. Conover , J. Campisi , J. M. van Deursen , Science 2016, 354, 472. 27789842 10.1126/science.aaf6659 PMC5112585

[45] Y. Zhu , T. Tchkonia , H. Fuhrmann‐Stroissnigg , H. M. Dai , Y. Y. Ling , M. B. Stout , T. Pirtskhalava , N. Giorgadze , K. O. Johnson , C. B. Giles , J. D. Wren , L. J. Niedernhofer , P. D. Robbins , J. L. Kirkland , Aging Cell 2016, 15, 428. 26711051 10.1111/acel.12445 PMC4854923

[46] C. M. Roos , B. Zhang , A. K. Palmer , M. B. Ogrodnik , T. Pirtskhalava , N. M. Thalji , M. Hagler , D. Jurk , L. A. Smith , G. Casaclang‐Verzosa , Y. Zhu , M. J. Schafer , T. Tchkonia , J. L. Kirkland , J. D. Miller , Aging Cell 2016, 15, 973. 26864908 10.1111/acel.12458 PMC5013022

[47] O. Moiseeva , X. Deschênes‐Simard , E. St‐Germain , S. Igelmann , G. Huot , A. E. Cadar , V. Bourdeau , M. N. Pollak , G. Ferbeyre , Aging Cell 2013, 12, 489. 23521863 10.1111/acel.12075

[48] F. Olivieri , M. R. Rippo , F. Prattichizzo , L. Babini , L. Graciotti , R. Recchioni , A. D. Procopio , Immun. Ageing 2013, 10, 11. 23506673 10.1186/1742-4933-10-11 PMC3618188

[49] M. Fabbri , A. Paone , F. Calore , R. Galli , E. Gaudio , R. Santhanam , F. Lovat , P. Fadda , C. Mao , G. J. Nuovo , N. Zanesi , M. Crawford , G. H. Ozer , D. Wernicke , H. Alder , M. A. Caligiuri , P. Nana‐Sinkam , D. Perrotti , C. M. Croce , Proc. Natl. Acad. Sci. U. S. A. 2012, 109, E2110. 22753494 10.1073/pnas.1209414109 PMC3412003

[50] R. Chen , A. B. Alvero , D. A. Silasi , M. G. Kelly , S. Fest , I. Visintin , A. Leiser , P. E. Schwartz , T. Rutherford , G. Mor , Oncogene 2008, 27, 4712. 18408758 10.1038/onc.2008.112 PMC3041589

[51] A. Sculean , D. Nikolidakis , G. Nikou , A. Ivanovic , I. L. C. Chapple , A. Stavropoulos , Periodontol. 2000 2015, 68, 182. 25867987 10.1111/prd.12086

[52] X. Ding , H. Zhao , Y. Li , A. L. Lee , Z. Li , M. Fu , C. Li , Y. Yang , P. Yuan , Adv. Drug Delivery Rev. 2020, 160, 78. 10.1016/j.addr.2020.10.005 33091503

[53] M. Su , L. Ruan , X. Dong , S. Tian , W. Lang , M. Wu , Y. Chen , Q. Lv , L. Lei , Int. J. Biol. Macromol. 2023, 227, 472. 36549612 10.1016/j.ijbiomac.2022.12.148

[54] X. Xing , H. Huang , X. Gao , J. Yang , Q. Tang , X. Xu , Y. Wu , M. Li , C. Liang , L. Tan , L. Liao , W. Tian , ACS Appl. Mater. Interfaces 2022, 14, 3885. 35014784 10.1021/acsami.1c22138

[55] J. Zhu , S. Yang , Y. Qi , Z. Gong , H. Zhang , K. Liang , P. Shen , Y. Huang , Z. Zhang , W. Ye , L. Yue , S. Fan , S. Shen , A. G. Mikos , X. Wang , X. Fang , Sci. Adv. 2022, 8, eabk0011. 35353555 10.1126/sciadv.abk0011 PMC8967232

[56] Z. Chen , Z. Lv , Y. Zhuang , Q. Saiding , W. Yang , W. Xiong , Z. Zhang , H. Chen , W. Cui , Y. Zhang , Adv. Mater. 2023, 35, 2300180. 10.1002/adma.202300180 37230467

[57] J. Bian , F. Cai , H. Chen , Z. Tang , K. Xi , J. Tang , L. Wu , Y. Xu , L. Deng , Y. Gu , W. Cui , L. Chen , Nano Lett. 2021, 21, 2690. 33543616 10.1021/acs.nanolett.0c04713

[58] S. Jin , Y. Wang , X. Wu , Z. Li , L. Zhu , Y. Niu , Y. Zhou , Y. Liu , Adv. Mater. 2023, 35, 2211602. 10.1002/adma.202211602 36779444

[59] D. Khorsandi , A. Fahimipour , P. Abasian , S. S. Saber , M. Seyedi , S. Ghanavati , A. Ahmad , A. A. De Stephanis , F. Taghavinezhaddilami , A. Leonova , R. Mohammadinejad , M. Shabani , B. Mazzolai , V. Mattoli , F. R. Tay , P. Makvandi , Acta Biomater. 2021, 122, 26. 33359299 10.1016/j.actbio.2020.12.044

[60] G. Wu , W. Wu , Q. Zheng , J. Li , J. Zhou , Z. Hu , Biomed. Eng. Online 2014, 13, 97. 25038793 10.1186/1475-925X-13-97 PMC4112644

[61] W. Huang , Q. Zheng , W. Sun , H. Xu , X. Yang , Int. J. Pharm. 2007, 339, 33. 17412538 10.1016/j.ijpharm.2007.02.021

[62] S. Chen , S. K. Boda , S. K. Batra , X. Li , J. Xie , Adv. Healthcare Mater. 2018, 7, 1701024. 10.1002/adhm.201701024 PMC5867260 29210522

[63] Y. Zhao , J. Yan , J. Yu , B. Ding , Macromol. Rapid Commun. 2023, 44, 2200740. 10.1002/marc.202200740 36271746

[64] E. Bellu , S. Cruciani , G. Garroni , F. Balzano , R. Satta , M. A. Montesu , A. Fadda , M. Mulas , G. Sarais , P. Bandiera , C. Ventura , M. Kralovič , J. Sabo , E. Amler , M. Maioli , Cells 2021, 10, 1415. 34200247 10.3390/cells10061415 PMC8227046

[65] T. Xu , Y. Cai , X. Zhong , L. Zhang , D. Zheng , Z. Gao , X. Pan , F. Wang , M. Chen , Z. Yang , Chem. Commun. 2019, 55, 7175. 10.1039/c9cc03056e 31162503

